# The Association of Maternal Lifestyle with Birth Defects in Shaanxi Province, Northwest China

**DOI:** 10.1371/journal.pone.0139452

**Published:** 2015-09-30

**Authors:** Leilei Pei, Yijun Kang, Yue Cheng, Hong Yan

**Affiliations:** 1 Department of Epidemiology and Health Statistics, School of Public Health, Xi’an Jiaotong University Health Science Center, Xi’an, Shaanxi, P.R. China; 2 Department of Nutrition and Food Safety Research, School of Public Health, Xi’an Jiaotong University Health Science Center, Xi’an, Shaanxi, P.R. China; Tabriz University of Medical Sciences, ISLAMIC REPUBLIC OF IRAN

## Abstract

**Background:**

The main objective was to investigate the burden of birth defects among alive infants and explore the impact of maternal lifestyle during pregnancy on the burden of birth defects in Northwest China.

**Methods:**

A stratified multi-stage sampling method was used to study infants born during 2010–2013 (and their mothers) in Shaanxi province of Northwest China. Socio-demographic information was collected using a structured questionnaire, and medical records from the local hospitals were used to determine the final diagnosis of birth defects. Poisson regression analysis was performed to assess the association between maternal lifestyles during pregnancy and the burden of birth defects, while adjusting for potential confounders.

**Results:**

We sampled 29098 infants, of whom 629 (i.e. 216.17 per 10000) were observed to have congenital defects. Cardiovascular system defects (77.32 per 10000) were found to be the most common. Mothers who had ever consumed alcohol during pregnancy were found to have infants with a higher prevalence of some categories of birth defects, including nervous system (Prevalence Rate Ratio, PRR:14.67, 95%CI:1.94, 110.92), cardiovascular system (PRR:3.22, 95%CI: 1.02, 10.16) and oral clefts (PRR:9.02, 95%CI: 2.08, 39.10) in contrast to infants of mothers without any alcohol consumption. Maternal passive smoking during pregnancy lead to the increased burden of malformations of eye, ear, face and neck (PRR:1.95, 95%CI:1.15, 3.33), cardiovascular system (PRR:1.70, 95%CI: 1.25, 2.31) and respiratory system (PRR:9.94, 95%CI: 2.37, 41.76) in their newborns. Further, tea or coffee consumption during pregnancy was positively correlated with the burden of specific birth defects, such as cardiovascular system (PRR: 2.44, 95%CI: 1.33, 4.46) and genital organs (PRR:14.72, 95%CI: 1.87, 116.11) among infants.

**Conclusions:**

The prevalence of birth defects was high in Shaanxi province of Northwest China. The unhealthy lifestyles of mothers during pregnancy may increase the prevalence of congenital malformations. These findings in future may have some important implications for prevention of birth defects in Northwest China.

## Introduction

Birth defects (BD), defined as a constellation of structural, functional and metabolic disorders, represent a significant global public health problem owing to their substantial contribution to infant and child mortality and morbidity. Around the world, an estimated 3% of the 134 million annual births are thought to be affected by birth defects, with significant variability in their prevalence among countries and regions [1]. The prevalence of birth defects ranges from 40 per 1000 live births in high-income countries to a maximum of 82 per 1000 live births in low-income countries [2]. Although, environmental and behaviors components are known to play a role in the etiology of birth defects, much ambiguity continues to persist around this subject [3–5]. The role of maternal lifestyle-related factors such as smoking and alcohol intake in the causation of certain categories of congenital defects is well established [6–8].

With the progressive decline in infant mortality in China, BDs have become one of the dominant problems of the country in recent years. Every year, an estimated 800,000 to 1.2 million infants are born with BDs and disabilities in China, accounting for 4%–6% of the country’s total newborns [9]. Simultaneously, the economic development is extremely unbalanced among different regions of China, with lower socio-economic level in Northwest China. According to the sixth census (2010) data in China, Shaanxi province of Northwest China had a population of approximately 37 million, and a fertility rate of 9.73‰. The impact of maternal lifestyles during pregnancy on the burden of birth defects among infants in Northwest China has not been adequately researched. Understanding of the relationship between maternal lifestyles during pregnancy and BDs can inform policy making and programming for birth defects prevention interventions. In 2013, a large cross-sectional population-based epidemiological survey of birth defects among infants was conducted in Shaanxi province, Northwest China. In the present study, we assessed the burden of birth defects among alive infants, and investigated the impact of maternal lifestyle during pregnancy on the burden of BDs in Northwest China. To the best of our knowledge, this is the first systematic investigation into the relationship between maternal lifestyle during pregnancy and birth defects based on a large population survey. This study is an attempt to fill an important knowledge gap in this geographical region.

## Materials and Methods

### Study design and participants

The study employed data from the cross-sectional survey conducted from August to November 2013 in Shaanxi province of Northwest China. The target population of the study were infants born during 2010–2013 and their mothers. Because of the differences in the distribution and fertility rates of population between rural and urban areas in the whole province, a stratified multi-stage sampling method was employed to determine the sampling units. In China, counties, townships and villages in rural areas and districts, streets and communities in urban areas, respectively, were two different three-level administration frames. As a first step, 20 counties and 10 districts were randomly selected from the entire province. In the next step, six townships in the chosen counties, and three streets in the chosen districts, were randomly sampled. Subsequently, six villages from each chosen township and six communities in the chosen streets were selected randomly. In the next stage, a random sampling method was adopted to select 30 babies born during 2010–2013 and their mothers in each sampled village, and, 60 in each chosen community. Approximately 32400 infants and their mothers were expected to be sampled in the study. However, 2374 subjects of the randomly sampled population declined to participate in the study (response rate: 92.7%). Only infants who were alive at the time of sampling were considered for this study. A total of 29098 live infants were eventually selected, and the relative sampling size in relation to the total infant population of the whole province was approximately 9%.

### Data collection

All family-level primary data was reported by the mothers of the sampled children in the pre-coded structured family questionnaire, including socio-demographical information, information on maternal lifestyles during pregnancy, and presence of birth defects at the time of visit. The information on birth defects was collected with the pre-coded structured birth defects questionnaire which contained questions on birth outcomes, past diagnostic information at local hospitals, time of diagnosis and the types of defects. In the survey, all questionnaires were designed by Xi’an Jiaotong University Health Science Center. Written informed consent was obtained from all subjects prior to their enrollment in the study. Families having a child were required to complete birth defects questionnaire. Medical records at local hospitals including prenatal diagnostic test results, clinic diagnostic results, findings of physical examination, ultrasound imaging reports and medical history were used as final diagnosis references.

Trained field staff from Xi’an Jiaotong University Health Science Center were responsible for the data collection. Ten investigation teams were formed for these counties or districts, with each team comprising of 10–12 members, a supervisor and a doctor from local maternal and child health hospital. As soon as each questionnaire was completed, the supervisors were under obligation to detect any errors and/or incomplete information. The doctor in each team facilitated the collection of information on birth defects. All data collection was completed in the local village clinics and community health service centers. The study was supported by the local hospitals and health administrative departments as well as the Shaanxi province Ministry of Health.

### Study variables

Birth defects were classified into 11 different groups based on the International Classification of Diseases, 10th Revision (ICD-10), including nervous system (Q00-Q07), eye, ear, face and neck (Q10-Q18), cardiovascular system (Q20-Q28), respiratory system (Q30-Q34), oral clefts (Q35-Q37), digestive system (Q38-Q45), genital organs (Q50-Q56), urinary system (Q60-Q64), musculoskeletal system (Q65-Q79), other defects (Q80-Q89), and chromosomal abnormalities (Q90-Q99). The mothers were interviewed in detail for their lifestyle during pregnancy (frequency of alcohol intake, passive smoking frequency, tea and coffee consumption). The alcohol intake frequency of mothers during pregnancy was divided into three categories (no, <1/week, ≥1/week). Passive smoking frequency was defined as the number of times when the respondent passively inhaled smoke for > 15 minutes per day, and similarly, also classified into three categories (no, <1/week, ≥ 1/week). The tea and coffee consumption during pregnancy had two dichotomous outcomes variables (no and yes). The Demographic and Health Survey wealth index based on the principal component analysis was adopted to assess the socioeconomic status (SES) of the households [10]. The first principal component representing the family economic level was classified as tertiles: poor, medium and rich. Other socio-demographic characteristics captured by the survey included infant gender (male or female), fetal number (singleton or twin and multi-fetal), residence during the pregnancy (permanent or floating), mother's education (no education, primary school, junior high school, senior high school, college and above), mother's age (< 25, 25–29 and ≥ 30 years groups), and parity (1, 2 and ≥ 3).

### Data analysis

Data were entered into Epidata 3.1 by double entry, and STATA version 12.0 (STATA Corp., TX, USA) was used for data analysis. The criteria for statistical significance was *P* value of < 0.05. Prevalence rates (per 10000 live births) of congenital malformations across socio-demographic characteristics and maternal lifestyles in the province were compared using univariate Poisson regression. Poisson regression model was also applied to assess the association between maternal lifestyles during pregnancy and birth defects after adjusting for socio-demographic characteristics. Prevalence rate ratio (PRR) was used as an indication of the magnitude of change, which was not be negative and <1 for a decrease and >1 for an increase.

### Ethics Statement

Written informed consent was obtained from all study participants after a detailed briefing on the purpose, process and confidentiality of the research. The study complied with the Declaration of Helsinki and was approved by the Ethics Committee of Xi’an Jiaotong University Health Science Center.

## Results

### Baseline characteristics of the participants

The average age of infants was 16.88 ± 11.29 months (range 0–46 months). Of the infants, 98.8% were singletons and boys accounted for 54.4% of total infants. The mean age of mothers was 28.01 ± 4.86 years and approximately 38.2% of them were educated beyond senior high school. Amongst the mothers, the average parity was 1.65 ± 0.82. Based on tertiles of household wealth index, there was an equal distribution of households in the poor, medium and rich category. The demographic profile of the study sample seemed to approximate that of the overall population of Shaanxi province based on the sixth Chinese population census (2010) data (Table 1). About 1.14% of mothers ever drank alcohol at least once per week during pregnancy, while more than 23.0% of them were exposed to passive smoking during pregnancy. Further, approximately 2.4% of the participants ever drank tea, and very few mothers consumed coffee during pregnancy. Data on maternal lifestyles during pregnancy disaggregated by socio-demographic characteristics are summarized in Table 2.

**Table 1:** Characteristics of the study population and the population of Shaanxi province ^a^.

Baseline characteristics	Study population	Population of Shaanxi province ^b^
**Parity**	1.46	1.05
**Infant Gender**		
Male	54.35	51.67
Female	45.65	48.33
**Mother's education**		
College and above	18.24	11.20
Senior high school	20.01	16.73
Junior high school	49.62	42.58
Primary school	10.2	24.84
No education	1.93	4.65
**Mother's age, years**		
< 25	24.84	26.90
25–29	42.31	10.45
≥ 30	31.29	62.65
**Residence during the pregnancy**		
Permanent	88.12	84.21
Floating	11.28	15.79
**Total**	29098	37327379

^a^ Data expressed as percentage or mean values.

^b^ Source: Sixth population census, China (2010).

**Table 2:** Maternal lifestyle during pregnancy by socio-demographic characteristics (N = 29098) ^a^.

	Alcohol consumption			Passive smokers			Tea consumption		Coffee consumption	
Baseline characteristics	no	< 1/week	≥ 1/week	no	< 1/week	≥ 1/week	no	yes	no	yes
**Infant Gender** *										
Male	15589(98.85)	111(0.70)	71(0.45)	11956(76.24)	759(4.84)	2968(18.92)*	15401(97.68)	366(2.32)	15654(99.28))	113(0.72)
Female	13098(98.87)	102(0.77)	48(0.36)	10244(77.87)	661(5.02)	2250(17.10)	12904(97.48)	334(2.52)	13133(99.18)	108(0.82)
**Fetal number**										
Singleton	28375(98.86)	211(0.74)	117(0.41)	21950(76.95)	1403(4.92)	5171(18.13)	27998(97.29)	691(2.71)	28472(99.23)	220(0.77)
Twin and multi-fetal	345(98.85)	2(0.57)	2(0.57)	275(79.25)	19(5.48)	53(15.27)	340(97.42)	9(2.58)	348(99.71)	1(0.29)
**Mother's education**										
College and above	5232(98.88)	41(0.77)	18(0.34)	4495(85.31)	267(5.07)	507(9.62)*	5193(98.22)	94(1.78)*	5230(98.90)	58(1.10)*
Senior high school	5733(98.74)	50(0.86)	23(0.40)	4648(80.78)	272(4.73)	834(14.49)	5659(97.52)	144(2.48)	5750(99.07)	54(0.93)
Junior high school	14228(98.90)	101(0.70)	57(0.40)	10739(75.05)	714(4.99)	2856(19.96)	13998(97.32)	385(2.68)	14283(99.30)	101(0.70)
Primary school	2927(98.89)	19(0.64)	14(0.47)	1962(66.92)	147(5.01)	823(28.07)	2894(97.84)	64(2.16)	2950(99.80)	6(0.20)
No education	551(98.57)	2(0.36)	6(1.07)	339(60.86)	21(3.77)	197(35.37)	548(98.03)	11(1.97)	557(99.64)	2(0.36)
**Mother's age, year**										
< 25	7128(98.75)	52(0.72)	38(0.53)	5450(76.27)	343(4.80)*	1353(18.93)*	7016(97.17)	204(2.83)*	7153(99.02)	71(0.98)*
25–29	12161(98.92)	89(0.72)	44(0.36)	9551(78.07)	643(5.26)	2040(16.67)	12032(97.92)	255(2.08)	12207(99.34)	81(0.66)
≥ 30	8992(98.91)	67(0.74))	32(0.35)	6870(75.99)	422(4.67)	1749(19.35)	8859(97.53)	224(2.47)	9018(99.30)	64(0.70)
**Parity** *										
1	16464(98.82)	122(0.73)	75(0.45)	13358(80.71)	789(4.77)	2404(14.52)*	16239(97.53)	412(2.47)	16495(99.02)	163(0.98)*
2	11106(98.91)	82(0.73)	40(0.36)	8081(72.37)	588(5.27)	2497(22.36)	10973(97.76)	252(2.24)	11170(99.55)	51(0.45)
≥ 3	998(99.20)	6(0.60)	2(0.20)	665(66.63)	42(4.21)	291(29.16)	980(97.42)	26(2.58)	1002(99.60)	4(0.40)
**Wealth index**										
Poor	9517(98.99)	65(0.68)	32(0.33)	7210(75.55)	421(4.41)	1912(20.04)*	9393(97.75)	216(2.25)	9562(99.53)	45(0.47)*
Medium	9533(98.83)	69(0.72)	44(0.46)	7431(77.53)	494(5.15)	1660(17.32)	9403(97.56)	235(2.44)	9568(99.26)	71(0.74)
Rich	9673(98.75)	79(0.81)	43(0.44)	7587(77.85)	507(5.20)	1652(16.95)	9545(97.46)	249(2.54)	9693(98.93)	105(1.07)
**Residence during the pregnancy**										
Permanent	3219(98.32)	41(1.25)	14(0.43)*	2607(79.85)	139(4.26)	519(15.90)*	3184(97.34)	87(2.66)	3233(98.81)	39(1.19)*
Floating	25344(98.94)	169(0.66)	102(0.40)	19496(76.65)	1278(5.02)	4660(18.32)	24999(97.64)	604(2.36)	25425(99.30)	180(0.70)
**Total**	28723(98.86)	213(0.73)	119(0.41)	22228(76.98)	1422(4.92)	5224(18.09)	28341(97.59)	700(2.41)	28823(99.24)	221(0.76)

^a^ Data expressed as frequency (percentage).

* indicates *P* < 0.05 for inter-group differences.

Compared to the floating population, the frequency of alcohol intake during pregnancy was higher in the permanently resident population. The older mothers with lower education and economic level were more likely to be passive smokers. With respect to coffee consumption in pregnancy, our study revealed a greater percentage in young and well educated women. In addition, low parity and better SES might also be two key contributing factors for higher coffee consumption among mothers.

### Prevalence of birth defects

The overall prevalence rate of congenital malformations was approximately 216.17 per 10000 alive infants. Cardiovascular system malformations had the highest prevalence (77.32 per 10000 alive infants), followed by other defects (38.15 per 10000 alive infants alive), musculoskeletal system defects (33.68 per 10000 alive infants), eye, ear, face and neck malformations (23.03 per 10000 alive infants), and oral clefts (11.68 per 10000 alive infants). The prevalence of birth defects appeared to have a significant correlation with socio-demographic characteristics such as infant’s gender, fetal number, etc. Among maternal factors, education level and age, parity, economic status and residence during pregnancy (Table 3) affected the prevalence of congenital malformations. We also observed an association of lifestyle-related factors (such as, alcohol intake, passive smoking and tea or coffee consumption), with the prevalence of different categories of congenital malformations (Table 4). There was a higher prevalence of birth defects of nervous system, oral clefts and urinary system among infants borne of mothers with a history of alcohol intake during pregnancy. Similarly, exposure to tobacco smoke during pregnancy was associated with a higher prevalence of birth defects involving eye, ear, face, neck, cardiovascular system and respiratory system. Mothers who ever consumed tea or coffee during pregnancy, appeared to have a link with the increased prevalence of cardiovascular system and genital organ malformations among the newborns.

**Table 3:** Prevalence of congenital malformations in infants by socio-demographic characteristics (N = 29098) ^a^.

Baseline characteristics	Nervous system	Eye, ear, face and neck	Cardiovascular system	Respiratory system	Oral clefts	Digestive system	Genital organs	Urinary system	Musculoskeletal system	Other defects	Chromosomal abnormalities	Total
Infant Gender*												
Male	13(8.23)	35(22.16)	131(82.94)	9(5.70)	24(15.19)	18(11.40)	18(11.40)	1(0.63)	59(37.35)	58(36.72)	5(3.17)	371(234.88)*
Female	12(9.04)	32(24.12)	94(70.85)	3(2.26)	10(7.54)	9(6.78)	1(0.75)*	2(1.51)	39(29.40)	53(39.95)	3(2.26)	258(194.47)
**Fetal number**												
Singleton	25(8.70)	65(22.61)	214(74.45)*	12(4.17)	33(11.48)	26(9.04)	16(5.57)*	3(1.04)	93(32.35)*	107(37.22)*	7(2.44)*0	601(209.07)*
Twin and multi-fetal	0(0.00)	2(57.31)	11(315.19)	0(0.00)	1(28.65)	1(28.65)	3(85.96)	0(0.00)	5(143.27)	4(112.61)	1(28.65)	28(802.29)
Mother's education*												
College and above	1(1.89)*	6(11.33)	45(84.94)	1(1.89)	5(9.44)	5(9.44)	2(3.78)	1(1.89)	6(11.33)*	16(30.20)	1(1.89)	89(167.99)*
Senior high school	4(6.88)	15(25.81)	41(70.54)	3(5.16)	2(3.44)	5(8.60)	2(3.44)	0(0.00)	14(24.09)	27(46.46)	1(1.72)	114(196.15)
Junior high school	10(6.94)	36(24.98)	112(77.72)	7(4.86)	21(14.57)	12(8.33)	13(9.02)	2(1.39)	64(44.41)	51(35.39)	4(2.78)	332(230.38)
Primary school	9(30.37)	7(23.62)	20(67.48)	1(3.37)	5(16.87)	5(16.87)	2(6.75)	0(0.00)	13(43.86)	16(53.98)	2(6.75)	80(269.91)
No education	1(17.86)	3(53.57)	6(107.14)	0(0.00)	1(17.86)	0(0.00)	0(0.00)	0(0.00)	1(17.86)	1(17.86)	0(0.00)	13(232.14)
Mother's age, years*												
< 25	3(4.15)	20(27.67)	44(60.87)*	4(5.53)	5(6.92)	8(11.07)	5(6.92)	1(1.38)	26(35.97)	26(35.97)	1(1.38)	143(197.84)*
25–29	14(11.37)	19(15.43)	91(73.92)	4(3.25)	17(13.81)	10(8.12)	6(4.87)	1(0.81)	38(30.87)	45(36.56)	3(2.44)	248(201.46)
≥ 30	7(7.69)	27(29.66)	87(95.56)	4(4.39)	12(13.18)	9(9.89)	8(8.79)	1(1.10)	31(34.05)	40(43.94)	4(4.39)	230(252.64)
Parity*												
1	8(4.80)*	32(19.18)*	118(70.74)*	7(4.20)	13(7.79)	12(7.19)	9(5.40)	2(1.20)	44(26.38)*	59(35.37)	4(2.40)	308(184.64)*
2	17(15.11)	28(24.89)	89(79.13)	5(4.45)	18(16.00)	14(12.45)	8(7.11)	1(0.89)	48(42.67)	46(40.90)	3(2.67)	277(246.27)
≥ 3	0(0.00)	6(59.64)	18(178.93)	0(0.00)	2(19.88)	1(9.94)	2(19.88)	0(0.00)	5(49.70)	6(59.64)	1(9.94)	41(407.55)
Wealth index*												
Poor	7(7.27)	31(32.20)*	85(88.28)	7(7.27)	13(13.50)	8(8.31)	8(8.31)	2(2.08)	39(40.51)	38(39.47)	3(3.12)	241(250.31)*
Medium	11(11.38)	18(18.63)	71(73.47)	3(3.10)	10(10.35)	11(11.38)	4(4.14)	0(0.00)	28(28.97)	45(46.56)	1(1.03)	202(209.02)
Rich	7(7.14)	18(18.36)	69(70.37)	2(2.04)	11(11.22)	8(8.16)	7(7.14)	1(1.02)	31(31.61)	28(28.55)	4(4.08)	186(189.68)
Residence during the pregnancy*												
Permanent	2(6.10)	10(30.49)	41(124.96)*	3(9.14)	6(18.29)	5(15.24)	1(3.05)	0(0.00)	16(48.77)*	20(60.96)*	2(6.10)	106(323.07)*
Floating	23(8.97)	57(22.23)	180(70.20)	9(3.51)	28(10.92)	22(8.58)	18(7.02)	3(1.17)	80(31.20)	89(34.71)	6(2.34)	515(200.85)

^a^ Data expressed as frequency (prevalence per 10000 live infants).

* denotes *P* < 0.05 for inter-group differences.

**Table 4:** Prevalence of type of congenital malformations by maternal lifestyle status during pregnancy ^a^.

		Alcohol consumption			Passive smokers			Tea consumption		Coffee consumption	
Groups of congenital malformations	All	no	< 1/week	≥ 1/week	no	< 1/week	≥ 1/week	no	yes	no	yes
Nervous system	25(8.59)	24(8.34)	0(0.00)	1(84.03)*	17(7.65)	2(14.06)	6(11.49)	25(8.80)	0(0.00)	25(8.66)	0(0.00)
Eye, ear, face and neck	67(23.03)	67(23.29)	0(0.00)	0(0.00)	43(19.34)	2(14.06)	22(42.11)*	66(23.24)	1(14.29)	67(23.20)	0(0.00)
Cardiovascular system	225(77.32)	220(76.48)	2(93.90)	3(252.10)	155(69.73)	9(63.29)	60(114.85)*	213(75.01)	12(171.43)*	223(77.22)	2(90.50)
Respiratory system	12(4.12)	12(4.17)	0(0.00)	0(0.00)	5(2.25)	3(21.10)	4(7.66)*	12(4.23)	0(0.00)	12(4.16)	0(0.00)
Oral clefts	34(11.68)	32(11.12)	2(93.90)	0(0.00)*	24(10.80)	1(7.03)	9(17.23)	33(11.62)	1(14.29)	34(11.77)	0(0.00)
Digestive system	27(9.28)	27(9.39)	0(0.00)	0(0.00)	19(8.55)	0(0.00)	8(15.31)	27(9.51)	0(0.00)	27(9.35)	0(0.00)
Genital organs	19(6.53)	19(6.61)	0(0.00)	0(0.00)	11(4.95)	2(14.06)	6(11.49)	19(6.69)	0(0.00)	18(6.23)	1(45.25)*
Urinary system	3(1.03)	2(0.70)	1(46.95)	0(0.00)*	2(0.90)	0(0.00)	0(0.00)	3(1.06)	0(0.00)	3(1.04)	0(0.00)
Musculoskeletal system	98(33.68)	97(33.72)	0(0.00)	1(84.03)	70(31.49)	6(42.19)	21(40.20)	95(33.45)	3(42.86)	96(33.24)	2(90.50)
Other defects	111(38.15)	110(38.24)	1(46.95)	0(0.00)	81(36.44)	7(49.23)	23(44.03)	110(38.74)	1(14.29)	110(38.09)	1(45.25)
Chromosomal abnormalitiesabnormalitiesabnormalities	8(2.75)	8(2.78)	0(0.00)	0(0.00)	5(2.25)	0(0.00)	3(5.74)	7(2.46)	1(14.29)	8(2.77)	0(0.00)
Total	629(216.17)	618(214.84)	6(281.69)	5(420.17)	432(194.35)	32(225.04)	162(310.11)*	610(214.80)	19(271.43)	623(215.74)	6(271.49)

^a^ Data expressed as frequency (prevalence per 10000 live infants).

* denotes *P* < 0.05 for inter-group differences.

### Predictors of birth defects

After adjusting for socio-demographic characteristics and other lifestyle-related variables during pregnancy, analysis based on Poisson model revealed a higher prevalence rate ratio (PRR) of certain categories of birth defects such as, nervous system (PRR:14.67, 95%CI: 1.94, 110.92), cardiovascular system (PRR:3.22, 95%CI: 1.02, 10.16) and oral clefts (PRR:9.02, 95%CI: 2.08, 39.10) among live infants born to women with a history of alcohol intake in pregnancy as compared to those born to mothers without a history of alcohol intake during pregnancy (Table 5). Further, there was a significantly increased PRR of malformations of eye, ear, face and neck (PRR:1.95, 95%CI: 1.15, 3.33), cardiovascular system (PRR:1.70, 95%CI: 1.25, 2.31) and respiratory system (PRR:9.94, 95%CI: 2.37, 41.76) among infants borne of mothers who ever passively smoked during pregnancy. In addition, tea or coffee consumption during pregnancy substantially increased the PRR for cardiovascular system (PRR: 2.44, 95%CI: 1.33, 4.46) and genital organs (PRR:14.72, 95%CI: 1.87,116.11) defects in offspring. However, no associations was observed between maternal history of alcohol, tea or coffee intake during pregnancy and the overall prevalence of congenital malformations, except for passive smoking (PRR: 1.53, 95%CI:1.27, 1.84).

**Table 5:** Association between congenital malformations and maternal lifestyles (N = 29098) ^a^ ^b^.

	Alcohol consumption			Passive smokers			Tea consumption		Coffee consumption	
Groups of congenital malformations	no	< 1/week	≥ 1/week	no	< 1/week	≥ 1/week	no	yes	no	yes
Nervous system	Ref	-	14.67(1.94, 110.92)*	Ref	1.77(0.40, 7.70)	1.20(0.46, 3.09)	Ref	-	Ref	-
Eye, ear, face and neck	Ref	-	-	Ref	0.74(0.18, 3.05)	1.95(1.15,3.33)*	Ref	0.74(0.10, 5.31)	Ref	-
Cardiovascular system	Ref	1.06(0.26, 4.33)	3.22(1.02, 10.16)*	Ref	0.85(0.42,1.73)	1.70(1.25,2.31)*	Ref	2.44(1.33,4.46)*	Ref	0.70(0.16,2.96)
Respiratory system	Ref	-	-	Ref	9.94(2.37,41.76)*	3.44(0.90,13.09)	Ref	-	Ref	-
Oral clefts	Ref	9.02(2.08,39.10)*	-	Ref	0.61(0.08,4.51)	1.17(0.51,2.65)	Ref	1.20(0.16,9.06)	Ref	-
Digestive system	Ref	-	-	Ref	-	1.75(0.76,4.07)	Ref	-	Ref	-
Genital organs	Ref	-	-	Ref	2.82(0.62,12.79)	2.07(0.75,5.72)	Ref	-	Ref	14.72(1.87,116.11)*
Urinary system	Ref	-	-	Ref	-	-	Ref	-	Ref	-
Musculoskeletal system	Ref	-	2.56(0.35,18.56)	Ref	1.18(0.47,2.92)	1.21(0.74,2.00)	Ref	0.87(0.20,3.74)	Ref	1.80(0.23,13.92)
Other defects	Ref	1.28(0.18,9.29)	-	Ref	1.35(0.62, 2.93)	1.17(0.73,1.89)	Ref	0.36(0.05,2.67)	Ref	1.64(0.22,12.27)
Chromosomal abnormalities	Ref	-	-	Ref	-	2.57(0.59,11.15)	Ref	7.08(0.87,57.81)	Ref	-
Any birth defects	Ref	1.06(0.44,2.58)	2.00(0.83,4.85)	Ref	1.11(0.77,1.61)	1.53(1.27,1.84)*	Ref	1.24(0.76,2.01)	Ref	1.00(0.40,2.48)

^a^ Poisson regression analysis of the association between birth defects and maternal lifestyle in pregnancy after adjusting for socio-demographic characteristics

^b^ Prevalence Rate Ratio (PRR) and 95% confidence interval are given to indicate the magnitude of change. Ref denotes reference group.

*P<0.05.

## Discussion

This large population-based cross-sectional study in Shaanxi province provides a representative picture of the burden of birth defects among alive infants, and explores the likely relationship between maternal lifestyles in pregnancy, and the burden of birth defects in the offspring. Results indicated a high overall prevalence of congenital malformations (approximately 216.17 per 10 thousand live infants) in Shaanxi province. Due to the cross-sectional study design, inferences on causal relationships between maternal lifestyle in pregnancy and the risk of birth defects in the offspring could not be drawn in our study. According to Poisson regression analysis, however, we inferred a potential association between a history of maternal passive smoking during pregnancy and the overall burden of congenital malformations. However, alcohol, tea and coffee intake did not demonstrate a significant association with the overall burden of congenital malformations. Likewise, significantly increased burden could be observed for different categories of congenital malformations among infants born to mothers with unhealthy lifestyles during pregnancy.

In our study, 629 infants of 29098 participants (216.17 per10000) were identified as having congenital defects in Shaanxi province, Northwest China. The prevalence of congenital malformations appears to be considerably higher than those reported from other parts of China. In a study conducted in Inner Mongolia, China, for example, the prevalence rate of birth defects was 156.1 per 10000 births (95%CI: 146.3–165.8) [11]. The Gansu birth defects survey revealed an overall incidence of 154.06/10000 births [12]. In economically developed regions, much lower prevalence rates of birth defects have been reported. For instance, the reported prevalence of birth defects in Shenzhen and Beijing was 14.14% and 17.56%, respectively [4,13].

There is much variability in the figures reported from different countries. According to Centers for Disease Control and Prevention, birth defects affect one in every 33 babies in the United States [14]. Out of 883,184 live births in Korea in 2005–2006, 25,335 were born with a birth defect, translating to a prevalence rate of 286.9 per 10,000 live births [15]. In Western Australia 4.5% to 5.7% of all live births between 2005 and 2008 were affected by birth defects [16].

In the present study, cardiovascular system malformations were the most common (77.32 per 10000 live infants), which was at variance with the figures from other recent studies in China [4,17]. In Korea, the anomalies of the circulatory system are the most common birth defects accounting for 43.4% of all birth defects [15]. Among Jordanian infants born between 2000 and 2005, congenital heart disease (CHD) was the most common birth anomaly (47%) [18]. Our study reveals cardiovascular system anomalies as being the most common form of congenital malformations in Shaanxi province, Northwest China.

About two thirds of Chinese male adults are regular smokers, whereas few Chinese women smoke; therefore, women’s passive smoking is more pervasive [19]. Among the respondents in our study, approximately 23% were exposed to environmental tobacco smoke for > 15 minutes on at least 1 day per week. Especially among the less educated mothers, with older age and lower SES, the percentage of passive environmental tobacco exposure was more likely to be higher. The findings are similar to that from studies conducted in UK and USA [20, 21].

In the present study, maternal passive smoking during pregnancy showed a positive correlation with a higher burden of cardiovascular system, respiratory system and facial malformations among alive infants. Similarly, a significant positive association of maternal smoking with cardiovascular/heart defects (OR: 1.09, 95%CI: 1.02, 1.17); facial defects (OR:1.19, 95%CI: 1.06, 1.35); eye defects (OR: 1.25, 95%CI: 1.11, 1.40) was reported by Hackshaw for [8]. The specific biological mechanisms underlying the association between tobacco smoke and fetal development have been examined in several human and laboratory studies. For example, it is now known that many of the 7000 chemicals in tobacco smoke could cross the placental barrier and have a direct harmful effect on the unborn baby [22,23]. However, the detailed mechanism of such an effect is yet to be completely understood.

In the current study, alcohol intake in pregnancy was positively associated with the increased burden of congenital malformations, especially those of nervous system, cardiovascular system and oral cleft. Previous studies have demonstrated that high intakes of alcohol adversely affects blood vessels in adults, and especially in pregnant women [7,24]. Greater exposure to alcohol during pregnancy also contributed to a constellation of structural and developmental defects in the offspring. In animal studies, alcohol exposure has been shown to be strongly associated with excessive cell death, formation of free radicals, as well as causing disturbances in the folate metabolism [25]. It is conceivable that the teratogenic effects of alcohol on nervous system, cardiovascular system and oral cleft operate through some of these mechanisms.

We also observed an association between tea or coffee consumption during pregnancy and the burden of congenital malformations of cardiovascular system and genital organs. The potential effects of caffeine, a natural alkaloid found in tea and coffee, has attracted much attention with regard to its possible effects on birth outcomes. Due to the progressive decrease in maternal caffeine clearance over the course of the pregnancy [26], caffeine intake by the mother is more likely to cross the placental barrier [27,28]. It is believed that that increased catecholamine release caused by caffeine, may cause fetal-placental vasoconstriction, hypoxia with consequent adverse impact on fetal growth and development [29]. More research is needed to unravel other mechanisms modulating the harmful effects of tea/coffee.

The multi-stage stratified random sampling methodology adopted for this study is a strength. Moreover, the sample closely matched the general population of Shaanxi province in terms of socio-demographic characteristics. Secondly, the inclusion of 29098 live infants makes our study one of the largest on this subject in Shaanxi province as well as in Northwest China. Thus, the present findings are likely to have a high degree of generalizability to the overall province.

However, several limitations should be considered while interpreting our results. The cross-sectional nature of study precluded drawing any cause-effect relationship. Secondly, the information on lifestyles during pregnancy including alcohol intake, passive smoking, tea or coffee consumption were self-reported by mothers. Although recent studies have showed a high sensitivity for self-reported events in pregnancy, the self-reports tend to have low specificity in that some women may under-report [30,31]. Therefore, we cannot rule out the possibility of misclassification of our exposures. In particular, the issue of recall bias may be exacerbated by the time elapsed between the pregnancy and the maternal self-report. To maximize power and minimize bias, we made a conscious effort at employing systematic approaches in the study design. During maternal interviews, information was gathered through the use of a standardized questionnaire and detailed supporting material (e.g., calendars) were available to help participants provide complete and accurate responses. Besides, the survey instruments were tested in a pilot study before the procedures and the detailed interviewer guides were developed. Reporting accuracy was maximized for mothers through highly structured interviews by skilled field staff from Xi’an Jiaotong University Health Science Center and experienced doctors from local maternal and child health hospital who were blinded to the study hypotheses.

Thirdly, although the analysis was adjusted for a number of socio-demographic confounders, the influence of other unknown potential confounders such as maternal health status, dietary intake, reproductive history, family history and environmental risk exposure during pregnancy, etc. cannot be ruled out. Therefore, we cannot eliminate the possibility that at least some of the risk estimates in our study were slightly overestimated. This could have contributed to the significant risk estimates reflected in the wide confidence limits in our study.

## Conclusions

In summary, Shaanxi province of Northwest China appears to have a high burden of birth defects among alive infants. The socio-demographic characteristics had a significant linkage with maternal lifestyles during pregnancy. A substantial proportion of mothers appear to be exposed to passive smoking and its attendant ill effects. A significant increased burden for congenital malformations was observed in infants born to mothers with unhealthy lifestyles in pregnancy. Further investigations are required to understand the mechanisms underlying the impact of maternal lifestyles in pregnancy on the birth defects in the offspring.

## Supporting Information

S1 Dataset Birth defects in Shaanxi province of China. (SAV)
